# Tree planting has the potential to increase carbon sequestration capacity of forests in the United States

**DOI:** 10.1073/pnas.2010840117

**Published:** 2020-09-21

**Authors:** Grant M. Domke, Sonja N. Oswalt, Brian F. Walters, Randall S. Morin

**Affiliations:** ^a^Forest Service Northern Research Station, US Department of Agriculture, St. Paul, MN 55108;; ^b^Forest Service Southern Research Station, US Department of Agriculture, Knoxville, TN 37919;; ^c^Forest Service Northern Research Station, US Department of Agriculture, York, PA 17402

**Keywords:** carbon, climate, emissions, removals, forest inventory

## Abstract

Several initiatives have been proposed to mitigate forest loss and climate change through tree planting as well as maintaining and restoring forest ecosystems. These initiatives have both inspired and been inspired by global assessments of tree and forest attributes and their contributions to offset carbon dioxide (CO_2_) emissions. Here we use data from more than 130,000 national forest inventory plots to describe the contribution of nearly 1.4 trillion trees on forestland in the conterminous United States to mitigate CO_2_ emissions and the potential to enhance carbon sequestration capacity on productive forestland. Forests and harvested wood products uptake the equivalent of more than 14% of economy-wide CO_2_ emissions in the United States annually, and there is potential to increase carbon sequestration capacity by ∼20% (−187.7 million metric tons [MMT] CO_2_ ±9.1 MMT CO_2_) per year by fully stocking all understocked productive forestland. However, there are challenges and opportunities to be considered with tree planting. We provide context and estimates from the United States to inform assessments of the potential contributions of forests in climate change mitigation associated with tree planting.

Forest ecosystems are the largest terrestrial carbon (C) sink on Earth (1), and their management has been recognized as a cost-effective strategy for mitigating greenhouse gas emissions. In the United States, forestland represents nearly one-third of total land area (Fig. 1 *A* and *B*), and forests store more than three decades of carbon dioxide (CO_2_) emitted from economy-wide fossil fuels (2). The contribution of forestland to emissions offsets in the United States has remained relatively stable since 2005 despite steady declines in economy-wide CO_2_ emissions over that period (2). This suggests that the forest C sink in the United States, which is driven in large part by forest regrowth following harvest and natural disturbance (3, 4), is slowly diminishing (4–7).

**Fig. 1.**
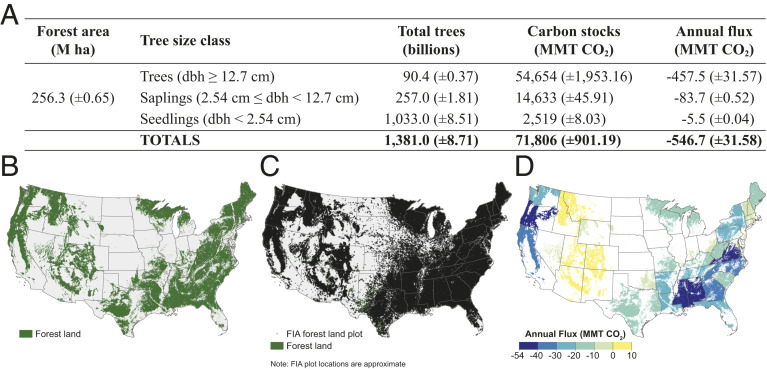
Estimates (with 95% CI) of (*A*) forestland area, number of trees, CO_2_ stocks, and annual flux by tree size class in the CONUS, and distribution of (*B*) forestland in the CONUS, (*C*) approximate locations of national forest inventory plots with at least one forested condition (n = 130,250) in the CONUS used in the study, and (*D*) total greenhouse gas emissions and removals on forestland by US state in 2018. Negative estimates indicate net C uptake (i.e., a net removal of C from the atmosphere).

Recently proposed afforestation and reforestation activities may accelerate live-tree sequestration of C stocks in forests (7, 8) and accumulation of C in soils (9), and potentially expand forestland (10), providing a multitude of ecosystem services (8). However, practical constraints and social and economic competition with other land uses and management objectives may limit implementation (5, 7). While uncertainty remains around climate change mitigation strategies, carbon markets have the potential to influence the priority placed on land management to promote forest C storage (5).

We use data from more than 130,000 national forest inventory (NFI) plots (Fig. 1*B*) to empirically describe the contribution of nearly 1.4 trillion trees on forestland in the conterminous United States (CONUS) to emissions offsets as well as opportunities and challenges to further enhance sequestration capacity. Specifically, we 1) describe the current status and extent of forestland in the CONUS, 2) characterize the current forestland C sink in the CONUS relative to economy-wide CO_2_ emissions (non-CO_2_ gases were not included in this study), and 3) highlight opportunities and challenges for increasing C sequestration capacity on existing forestland.

This work provides context and estimates for assessments of the potential contributions of trees and forests to mitigate forest loss and climate change through tree planting in the United States.

## Results

There are an estimated 1.38 trillion live trees (±8.71 billion live trees, 95% CI) across all size classes on 256.3 Mha of forestland (±0.65 Mha) in the CONUS (Fig. 1*A*). Collectively, there are an estimated 71,808 million metric tons (MMT) carbon dioxide (CO_2_) (±901.19 MMT CO_2_) stored in all live trees (aboveground and belowground) and they sequestered an estimated 546.7 MMT CO_2_ (±31.6 MMT CO_2_) in the year 2018 (Fig. 1 *A* and *D*). The CONUS-wide estimates translate to 280 MT CO_2_ stored per hectare across forestland in the CONUS with annual net sequestration of 2.13 MT CO_2_⋅ha^−1^⋅y^−1^.

**Fig. 2.**
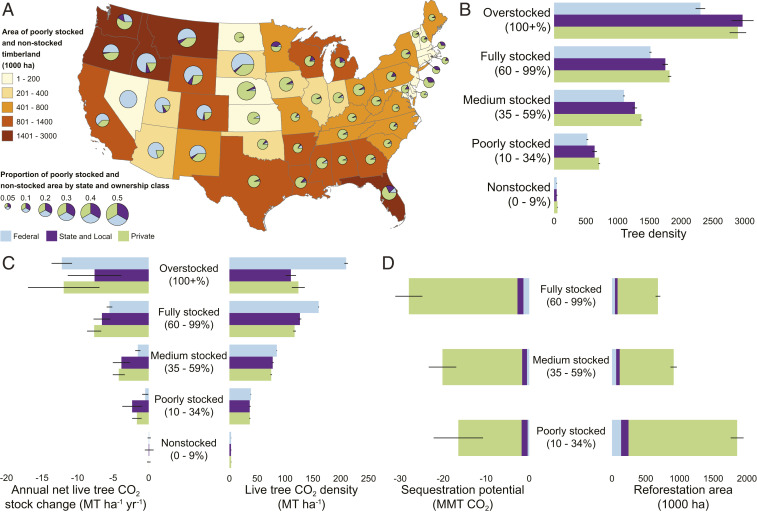
Distribution of (*A*) understocked timberland by ownership in the CONUS, (*B*) tree density by ownership and all live stocking on timberland in the CONUS (number of trees), (*C*) aboveground live-tree CO_2_ density and mean annual net CO_2_ flux by ownership and all live-tree stocking in the CONUS, and (*D*) reforestation area and CO_2_ sequestration potential—based on current tree planting capacity in the CONUS—when increasing stocking on timberland from nonstocked to poorly stocked, medium stocked, or fully stocked. Error bars represent the 95% CI. Negative estimates indicate net C uptake (i.e., a net removal of C from the atmosphere).

There are opportunities on existing forestland to increase the contribution of forests to climate change mitigation. Nearly 33 Mha (±0.47 Mha) of productive forestland (i.e., timberland) is classified as nonstocked or poorly stocked (<35% of the forestland area is occupied by trees; hereafter referred to as understocked) with live trees and seedlings (Fig. 2*A*). A disproportionate amount (44%, 14.5 Mha) of the understocked timberland is in the western states, which only represents 24% (49.1 Mha) of the total timberland land area in the United States. The understocked timberlands in the United States store less than 30% of the aboveground live-tree CO_2_ per unit area that fully stocked forests store (Fig. 2 *B* and *C*), and the sequestration capacity is substantially diminished—less than 20% of fully stocked forests—due to the limited area occupied by trees (Fig. 2 *B* and *C*).

Currently, there is federal infrastructure to produce and plant ∼65 million seedlings per year, and state and private capacity is ∼1.1 billion tree seedlings per year (11). Collectively, the estimated 1.2 billion trees planted on forestland sequester between 16 MMT CO_2_ and 28 MMT CO_2_ each year (Fig. 2*D*). Spatially concentrating current tree planting capacity to fully stock nonstocked timberland, rather than planting the same number of trees over larger areas, provides the greatest potential to increase C sequestration capacity, particularly on private timberland (Fig. 2*D*). In addition, increasing tree planting capacity to fully stock timberlands can potentially reduce the current reforestation backlog on federal forestland, increase total forestland sequestration capacity, and contribute to C storage (Fig. 2).

## Discussion

The contribution of existing forestland and harvested wood products to climate change mitigation in the United States is unmistakable (1, 5, 6); however, the sink has remained relatively stable, while total economy-wide CO_2_ emissions in the United States have declined (2). Considering trends in natural and anthropogenic disturbances (5), declines in forest regrowth are likely to continue in the absence of forest management (5, 10).

Tree planting may accelerate live-tree sequestration of C stocks in forests (7, 8) and the accumulation of C in soils (9). However, infrastructural constraints (e.g., planting stock availability), as well as social and economic competition with other land uses and management objectives (5, 7), natural disturbances (e.g., wildfire), and climate change (4, 5), have limited and may continue to limit implementation. Approximately 1% of understocked federal timberland is reforested each year, despite mandates requiring reforestation (7). Current tree planting efforts contribute ∼3 to 5% to live-tree C sequestration each year in the United States. If all understocked timberland were fully stocked in the United States, potential C sequestration capacity would increase by ∼20% (−187.7 MMT CO_2_ ±9.1 MMT CO_2_) per year, and immediate opportunities exist to build infrastructure and use resources from tree planting initiatives to restore and improve forest ecosystems (7).

This study provides context and empirical estimates from existing forestland in the CONUS. While we focused on reforestation and supplemental planting on understocked timberland, there are more than 168 Mha of other public and private timberland in the CONUS which may benefit from forest management activities. Further, there may be opportunities on land which was historically forested (reforestation) or where the current or past land use was not forestland (afforestation) (12). Finally, while reforestation and afforestation activities will help to maintain and potentially enhance the forest C sink in the United States and beyond (12), this is just one of many nature-based solutions which must be deployed to mitigate climate change.

## Methods

This analysis relied on the most recent publicly available data from the US NFI conducted by the US Department of Agriculture (USDA) Forest Service Forest Inventory and Analysis (FIA) program (13). Base intensity permanent ground plots are distributed approximately every 2,428 ha across the CONUS. Each permanent ground plot is a series of four fixed-radius (7.32 m) plots (i.e., subplots) spaced 36.6 m apart in a triangular arrangement with one subplot in the center. Tree-level (diameter at breast height [dbh] ≥ 12.7 cm) and site-level attributes are measured at regular temporal intervals on plots that have at least one forested condition. Saplings (2.54 cm ≤ dbh < 12.7 cm) and seedlings (dbh < 2.54 cm, conifer height ≥ 15.24 cm; hardwood height ≥ 30.48 cm) were measured and counted, respectively, on fixed-radius (2.07 m) microplots nested within each subplot.

All seedlings and live trees with a dbh ≥ 2.54 cm on forestland in the CONUS were included in this study. Population and ratio estimates of trees and seedlings, forestland area, all live-tree stocking, and C density and sequestration capacity (and associated uncertainties) were obtained following methods described in Bechtold and Patterson (14), US Environmental Protection Agency (EPA) (2), and USDA Forest Service (15). The estimates of seedling C stocks and flux include understory vegetation (15). Estimates of state and national C flux on forestland were obtained following methods in the US EPA (2).

Replanting scenarios were based on USDA Forest Service estimates of current annual tree planting capacity (11), and reforestation estimates were based entirely on empirical estimates obtained from the NFI and current tree planting capacity (11, 13–15).

## Data Availability

National forest inventory data have been deposited in FIA DataMart (https://apps.fs.usda.gov/fia/datamart/).

## References

[1] Pan Y.., A large and persistent carbon sink in the world’s forests. Science 333, 988–993 (2011). 21764754 10.1126/science.1201609

[2] US Environmental Protection Agency, “Inventory of U.S. Greenhouse Gas Emissions and Sinks: 1990-2018” (EPA 430-R-20-002, Environmental Protection Agency, 2020).

[3] Pugh T. A. M.., Role of forest regrowth in global carbon sink dynamics. Proc. Natl. Acad. Sci. U.S.A. 116, 4382–4387 (2019). 30782807 10.1073/pnas.1810512116 PMC6410874

[4] Williams C. A., Gu H., MacLean R., Masek J. G., Collatz G. J., Disturbance and the carbon balance of US forests: A quantitative review of impacts from harvests, fires, insects, and droughts. Global Planet. Change 143, 66–80 (2016).

[5] Domke G.., “Forests” in Second State of the Carbon Cycle Report (SOCCR2): A Sustained Assessment Report, Cavallaro N., Ed. (US Global Change Research Program, Washington, DC, 2018), pp. 365–398.

[6] Domke G. M., “Greenhouse gas emissions and removals from forest land, woodlands, and urban trees in the United States, 1990-2018” (Resource Update FS-227, US Department of Agriculture, Madison, WI, 2020).

[7] Nave L. E.., The role of reforestation in carbon sequestration. New For. 50, 115–137 (2019).

[8] Law B. E.., Land use strategies to mitigate climate change in carbon dense temperate forests. Proc. Natl. Acad. Sci. U.S.A. 115, 3663–3668 (2018). 29555758 10.1073/pnas.1720064115 PMC5889652

[9] Nave L. E.., Reforestation can sequester two petagrams of carbon in US topsoils in a century. Proc. Natl. Acad. Sci. U.S.A. 115, 2776–2781 (2018). 29483245 10.1073/pnas.1719685115 PMC5856546

[10] Birdsey R., Pregitzer K., Lucier A., Forest carbon management in the United States: 1600-2100. J. Environ. Qual. 35, 1461–1469 (2006). 16825466 10.2134/jeq2005.0162

[11] Haase D. L., Forest nursery seedling production in the United States—Fiscal year 2018. Tree Planters’ Notes 62, 20−24 (2019).

[12] National Academies of Sciences, Engineering, and Medicine, Negative Emissions Technologies and Reliable Sequestration: A Research Agenda, (The National Academies Press, Washington, DC, 2020). 31120708

[13] USDA Forest Service, The Forest Inventory and Analysis Database. Version 8.0. https://www.fia.fs.fed.us/tools-data/. Accessed 7 May 2020.

[14] Bechtold W. A., Patterson P. L., “The enhanced forest inventory and analysis program-national sampling design and estimation procedures” (Gen. Tech. Rep. SRS-80, US Department of Agriculture, Asheville, NC, 2005).

[15] USDA Forest Service, Database Description User Guide for Phase 2. Version 8.0. https://www.fia.fs.fed.us/library/database-documentation/current/ver80/FIADB%20User%20Guide%20P2_8-0.pdf. Accessed 7 May 2020.

